# Size Effect and Crystal–Melt Interfacial Tension in the Formation of Alloys from Ultrafine Sublayers of Dissimilar Metals

**DOI:** 10.3390/nano16201303

**Published:** 2026-10-15

**Authors:** Valeriy Volodin, Yuriy Tuleushev, Yeldar Zhakanbayev, Bakytzhan Tynyshbay

**Affiliations:** Institute of Nuclear Physics, Satbayev University, Almaty 050032, Kazakhstan; volodinv_n@mail.ru (V.V.); yuriy.tuleushev@mail.ru (Y.T.); zhakanbayev.yeldar@inp.kz (Y.Z.)

**Keywords:** ultrathin sublayers, magnetron sputtering, alloy formation, crystal–melt interface, interfacial tension, thermodynamics

## Abstract

Thirty-four binary systems were prepared by co-sputtering dissimilar metals onto a moving substrate. In the present work, the experimentally controlled size parameter is the nominal thickness of a single metal sublayer deposited during one substrate passage rather than a directly measured nanoparticle diameter. A formal linear fit between the characteristic sublayer thickness and the bulk melting temperature gives the empirical relation $d_{\mathrm{Me}}=1.46-7.3\times 10^{-5}T_{0}$, with a low coefficient of determination, $R^{2}=0.007$, indicating that the bulk melting temperature alone explains only a small part of the observed variation. Alloy composition and deposition conditions also affect the characteristic sublayer thickness. Using the Thomson equation within an effective-size approximation, the crystal–melt interfacial tension of the constituent metals was estimated. The approach provides a practical framework for selecting deposition conditions for the synthesis of binary alloy systems, while the calculated interfacial-tension values should be regarded as model-based estimates.

## 1. Introduction

The formation of materials by nanoscale deposition and the behavior of ultrafine metallic systems have attracted considerable research interest. Many studies have examined the size dependence of the melting temperature of small particles and clusters [1,2,3,4,5,6,7,8,9,10,11,12].

In Ref. [1], the generalized Thomson formula for the melting temperature of a small object is analyzed from the perspective of thermodynamic similarity. However, the available experimental data remain insufficient to draw a reliable conclusion regarding the accuracy of melting-temperature predictions for metal nanoparticles. In Ref. [2], a three-phase model of a simple substance and a nanocrystal model were used to derive an expression for the Helmholtz free energy, which accounts for both lattice vacancies and interstitial atoms. It was shown that for a certain number of atoms in the nanosystem, the S-shaped hysteresis loop of the crystal–liquid phase transition disappears; below this number, the transition does not occur. Various authors have modeled the melting-point depression of Sn and Pb [3], Al [4], V and Ni [5,6], Au, Pd and Pt [7,8], silver [9], and copper [10] using different approaches. The author of Ref. [11] calculated the melting points of nanoparticles of 48 metals and found that most 1 nm particles are unstable at room temperature.

Numerous studies have focused on the thermodynamics of small systems. In Refs. [12,13,14], the heat of melting of nanoparticles, the vacancy formation energy, and the prediction of phase transitions were examined. It was found that at a certain number of atoms, the hysteresis between the melting point and the crystallization onset temperature vanishes, and that the vacancy concentration increases with decreasing particle size. The authors of Refs. [15,16,17] extensively modeled the thermodynamic functions of nanocrystal melting and found that both the enthalpy and entropy of melting decrease with decreasing nanoparticle size. Moreover, surface atoms dominate the size-dependent effects on thermodynamic properties.

The size dependences and thermodynamic functions of melting have been studied for Ag, Pb and Al in Ref. [18], for Fe, Sn, Ag and In in Ref. [19], for Si in Ref. [20], for Sn, Ag, Cu and In in Ref. [21], and for Cu in Ref. [22].

In contrast to single-component nanoscale systems, bimetallic systems exhibit additional effects associated with the interaction of two different elements, including interdiffusion, segregation, chemical ordering, and composition-dependent coalescence. These effects have been investigated in several representative binary systems. Goudeli and Pratsinis studied coalescing Ag–Au nanoparticles and showed that Ag and Au exhibit different atomic mobilities during sintering, accompanied by surface segregation effects [23]. Cu–Ni bimetallic systems have been investigated experimentally and by Monte Carlo simulation, including the formation of a neck between interacting particles and the temperature dependence of coalescence [24]. For Pt–Pd nanoalloys, magnetron-sputtering aggregation experiments demonstrated that the degree of coalescence can be controlled by the processing conditions [25]. The melting and coalescence behavior of the miscible Ni–Pt system was also investigated using classical molecular-dynamics simulations [26].

These studies demonstrate that the formation and coalescence of bimetallic nanoscale systems depend not only on size but also on composition, atomic mobility, chemical ordering, and synthesis conditions. However, most previous investigations have focused on individual binary systems. Systematic comparisons of a relatively broad set of dissimilar-element combinations produced using a common co-sputtering method and deposition geometry have received comparatively less attention.

In discussing the thermodynamics of small systems, Roduner [27] emphasized several key points: for sufficiently small systems, the concepts of a phase state and a phase transition lose their usual meaning; in very small isolated systems, fluctuations become dominant and a meaningful definition of temperature may become problematic; and the melting and boiling points of liquid nanodroplets are lower than the corresponding bulk values.

In our previous work [28], we examined 20 binary metastable alloys of non-ferrous and rare metals obtained by co-deposition of ultrafine components. When the results were considered in the context of the Hume–Rothery rules, which apply to equilibrium systems, we observed a substantial increase in the solubility of the second element in the matrix metal. In many cases, the 15% limit on the atomic-size difference was exceeded for metals with either similar or different lattice types. The valence criterion was also frequently not satisfied, while only isolated cases were consistent with the criterion based on differences in Pauling electronegativity.

The present study extends this approach by systematically comparing alloy-formation conditions for a relatively broad set of binary combinations prepared using the same co-sputtering method. The nominal thickness of a single metal sublayer deposited during one substrate pass is used as a reproducible characteristic deposition parameter. The experimental data are used to examine an empirical relationship between this characteristic sublayer thickness and the bulk melting temperature of the constituent metals. In addition, the Thomson equation is applied within an effective-size approximation to obtain model-based estimates of the crystal–melt interfacial tension. Thus, the specific contribution of this work is the comparative analysis of multiple binary systems prepared under common deposition conditions, followed by a thermodynamic analysis based on the experimentally determined sublayer-thickness parameter.

## 2. Methods and Calculations

Thirty-four binary systems of various compositions were produced by co-sputtering dissimilar metals onto a moving substrate. The total coating was built up by repeated deposition of ultrathin sublayers of each metal. In the present work, $d_{\mathrm{Me}}$ denotes the nominal thickness of a single metal sublayer deposited during one passage of the substrate through the corresponding sputtered-metal flux. This parameter is not interpreted as the directly measured geometrical diameter of an individual nanoparticle.

The alloys were prepared by simultaneous ion-plasma sputtering of two metal targets using magnetron sources mounted on opposite walls of a vacuum chamber, followed by deposition onto a moving substrate. A detailed description of the method for producing binary coatings from dissimilar metals is given in Ref. [29].

The nominal sublayer thickness of each metal was calculated from the total coating thickness and the number of substrate passages through the corresponding sputtered-metal flux. The relevant controlled parameters included the deposition time, the number of substrate passages, and the magnetron sputtering power. Because the two metals were sputtered from oppositely positioned magnetrons, the amount of each deposited component could be controlled independently. By varying the deposition conditions, the nominal sublayer thicknesses were progressively changed.

Preliminary deposition experiments were carried out for each binary system with progressively decreasing sublayer thicknesses of the constituent metals. At relatively large sublayer thicknesses, X-ray diffraction revealed crystalline lattices characteristic of the individual metals. When a characteristic threshold thickness was reached, diffraction peaks corresponding to a solid-solution lattice were observed. The characteristic values reported in Table 1 correspond to the deposition conditions at which the transition to solid-solution formation was observed.

The deposition process proceeds through an island-type growth mechanism followed by coalescence. In this context, and following the analogy discussed by Roduner [27] between the melting-point depression of thin films and that of small particles formed through island growth, the nominal sublayer thickness is used as an effective characteristic length scale. Accordingly, values such as 0.1–0.2 nm represent nominal equivalent sublayer thicknesses rather than geometrical diameters of individual nanoparticles.

The substrate temperature during the experiments was approximately 373–423 K (100–150 °C). Under these conditions, conventional bulk solid-state diffusion is expected to be strongly limited. In our interpretation, the transition observed by X-ray diffraction from separate crystalline phases to a solid-solution lattice, together with the dependence on sublayer thickness, is consistent with enhanced atomic mobility and a possible quasi-liquid state of the ultrafine deposited material. When the sublayer thicknesses were larger than the characteristic threshold, complete coalescence was not observed and the same solid-solution structure was not detected. Nevertheless, the formation of a solid solution is not regarded as an unambiguous proof of a quasi-liquid state, and other mechanisms, including energetic-particle-assisted mixing, interfacial diffusion, defect-enhanced diffusion, and limited solid-state interdiffusion, may also contribute during magnetron sputtering.

**Figure 1:**
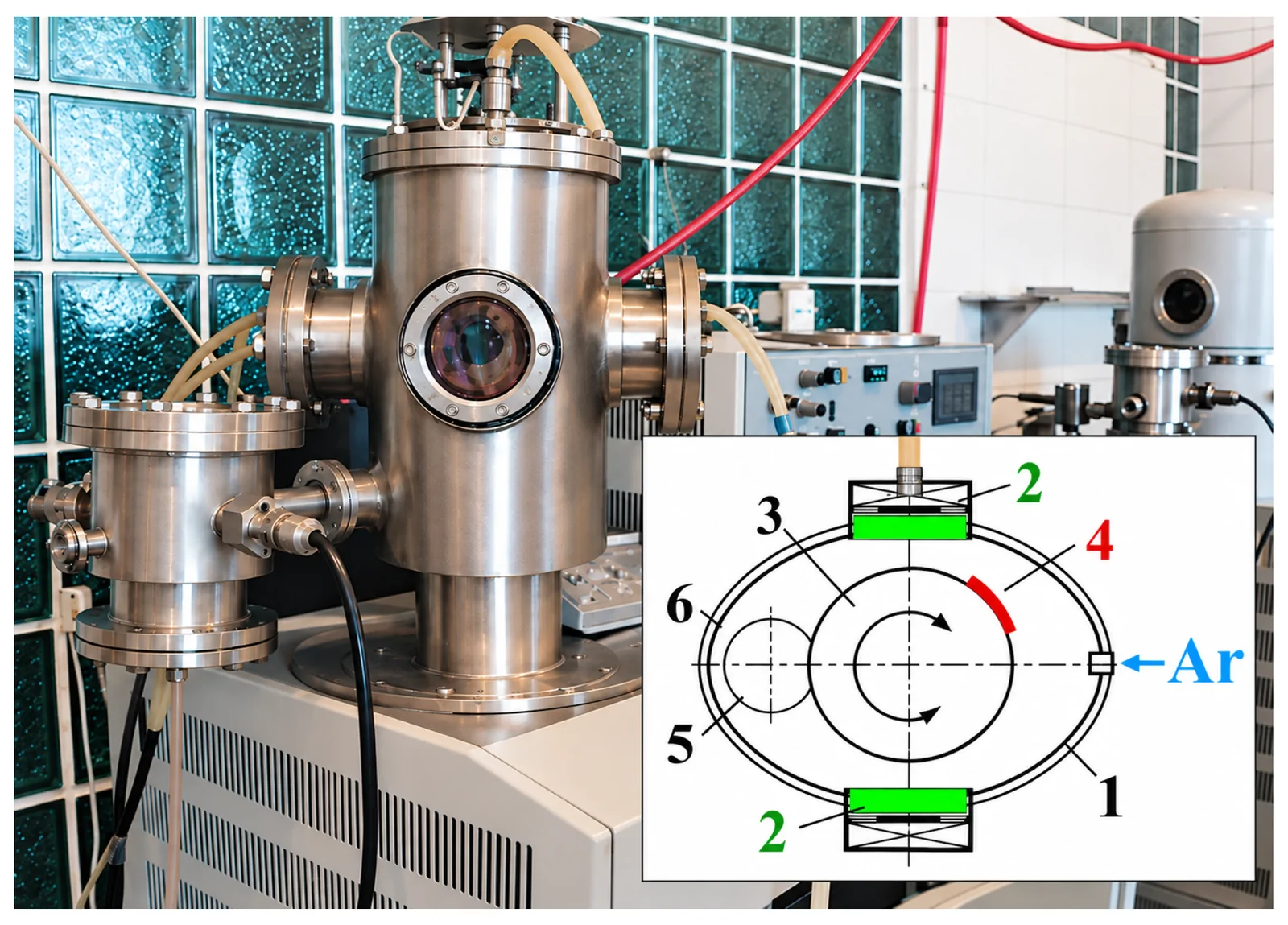
Scheme of the ion-plasma setup: 1—vacuum chamber body; 2—magnetrons; 3—cylinder; 4—substrate; 5—gas evacuation port; 6—housing.

The alloys were synthesized using a custom-designed ion-plasma system with two independent magnetron sputtering units, the schematic of which is shown in Figure 1.

The setup consists of a vacuum chamber containing planar DC magnetrons equipped with water-cooled targets mounted on its walls. A rotatable cylinder is installed inside the chamber to hold the substrates and move them relative to the magnetrons. High-purity argon was used as the plasma-forming gas. The alloys were produced by co-sputtering dissimilar metals onto a substrate moving relative to the plasma fluxes. The targets were disks of the corresponding metals, 40 mm in diameter and 4 mm thick.

The concentration ratio of the metals in the samples was controlled through the target sputtering rates, i.e., by adjusting the electrical power supplied to the magnetrons. The ratio of the deposited components was monitored gravimetrically, based on the amounts of sputtered and deposited metal, and independently by Rutherford backscattering spectrometry (RBS) with protons using the UKP-2-1 tandem accelerator at the Institute of Nuclear Physics (Almaty).

Polycor (α-${\mathrm{Al}}_{2}O_{3}$) and single-crystal silicon were used as substrates for preparing solid-solution coatings. X-ray diffraction (XRD) analysis was performed using a Bruker D8 Advance diffractometer with Cu Kα radiation (λ=0.154051 nm) and a graphite monochromator. The lattice parameter was obtained by averaging the values calculated from all diffraction lines of the corresponding phase.

The melting temperature of nanoscale metallic systems may depend on several factors, including characteristic size, composition, crystal structure, the presence of other elements, and surface properties. In the present work, the nominal sublayer thickness $d_{\mathrm{Me}}$ is used as the characteristic length scale. The melting-point depression (ΔT) is approximated by the hyperbolic relation

(1) $$\Delta T=k\cdot \frac{1}{d_{\mathrm{Me}}},$$

where $d_{\mathrm{Me}}$ is the nominal sublayer thickness (m) and *k* is a coefficient with units of K·m. The coefficient is determined from the corresponding melting-point depression and the experimentally determined characteristic sublayer thickness as $k=\Delta T\;d_{\mathrm{Me}}=\left(T_{0}-T\right)d_{\mathrm{Me}}$. In the calculations below, 373 K is used as the reference substrate temperature. Because the characteristic sublayer thickness of a given metal varies among the investigated binary systems, the corresponding value of *k* also varies. For example, for the Ta–Cd system, the calculated range of *k* is approximately (5.79–49.2) $\times \;10^{-7}$ K·m for Ta and (4.64–8.84) $\times \;10^{-7}$ K·m for Cd. This relation is subsequently used to estimate the melting-point depression at an effective size of 100 nm and to analyze the size dependence of the calculated interfacial tension [30].

The crystal–melt interfacial tension was calculated from the Thomson equation, with the Laplace-pressure correction neglected because of its small magnitude:

(2) $$\Delta T=T_{0}-T=\frac{2\sigma T_{0}}{\Delta H\;\rho_{s}R_{\mathrm{eff}}},\;\sigma =\frac{\Delta T\;\Delta H\;\rho_{s}d_{\mathrm{Me}}}{4T_{0}},$$

where $T_{0}$ is the bulk melting point (K), *T* is the size-dependent melting temperature (K), ΔH is the latent heat of fusion per unit mass (J/kg), $\rho_{s}$ is the density of the crystalline metal (kg/$m^{3}$), and σ is the crystal–melt interfacial tension (N/m). Within the effective-size approximation used here, $R_{\mathrm{eff}}=d_{\mathrm{Me}}/2$. The density and heat-of-fusion values were taken from Ref. [31] and converted to J/kg where necessary.

## 3. Results and Discussion

The obtained binary alloys and the maximum nominal sublayer thicknesses at which coalescence and solid-solution formation were observed are presented in Table 1. Different values of $d_{\mathrm{Me}}$ for the components of the binary systems were obtained in separate experiments involving different metal combinations and solid solutions of different compositions.

**Figure 2:**
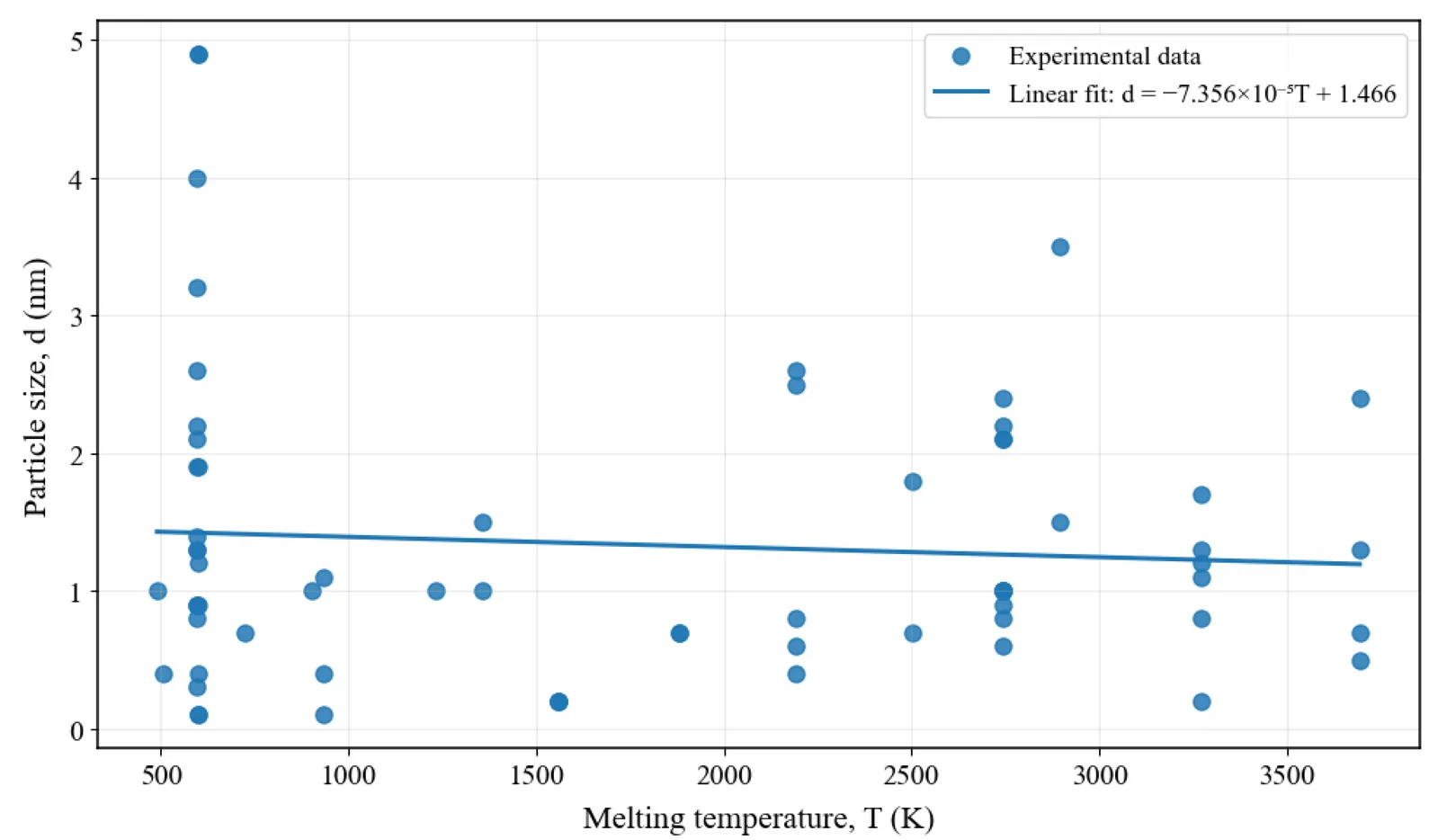
Dependence of the nominal metal sublayer thickness on the bulk melting temperature.

A formal evaluation of the dependence of the characteristic sublayer thickness on the melting temperature of the corresponding bulk metals, without taking other factors into account (Figure 2), gives the following empirical relationship:

(3) $$d_{\mathrm{Me}}=1.46-7.3\;\times 10^{-5}T_{0}.$$

The coefficient of determination is very small, $R^{2}=0.007$, confirming the considerable scatter of the experimental data and indicating that the bulk melting temperature alone explains only a small fraction of the variation in $d_{\mathrm{Me}}$. The observed scatter may be associated with differences in the total thickness of the binary films, deposition time, alloy composition, degree of deposition of the sputtered metals, variations in the applied magnetron power, and changes in the target sputtering rate during target erosion.

Nevertheless, the experimental dataset indicates a characteristic sublayer-thickness scale of approximately 1.46 nm for solid-solution formation. The contribution associated with the bulk melting-temperature term in Equation (3) is comparatively small; for example, for tungsten it is approximately 0.27 nm. Equation (3) should therefore be regarded as an approximate empirical trend of the experimental data rather than as a statistically strong or universal relationship.

The practical applicability of the magnetron-deposition approach is illustrated by related studies in which it was used to prepare and investigate V–Pb coatings, a new structural state of tungsten in porous tungsten coatings, and the intermetallic compound ${\mathrm{NbCd}}_{2}$ [32,33,34].

Using the characteristic nominal sublayer thickness estimated from Equation (3), the crystal–melt interfacial tension was calculated for each constituent metal within the effective-size approximation adopted in this work. The calculated interfacial tension, melting point, heat of fusion ($\Delta H_{\mathrm{mp}}$), and crystalline density are listed in Table 2.

**Figure 3:**
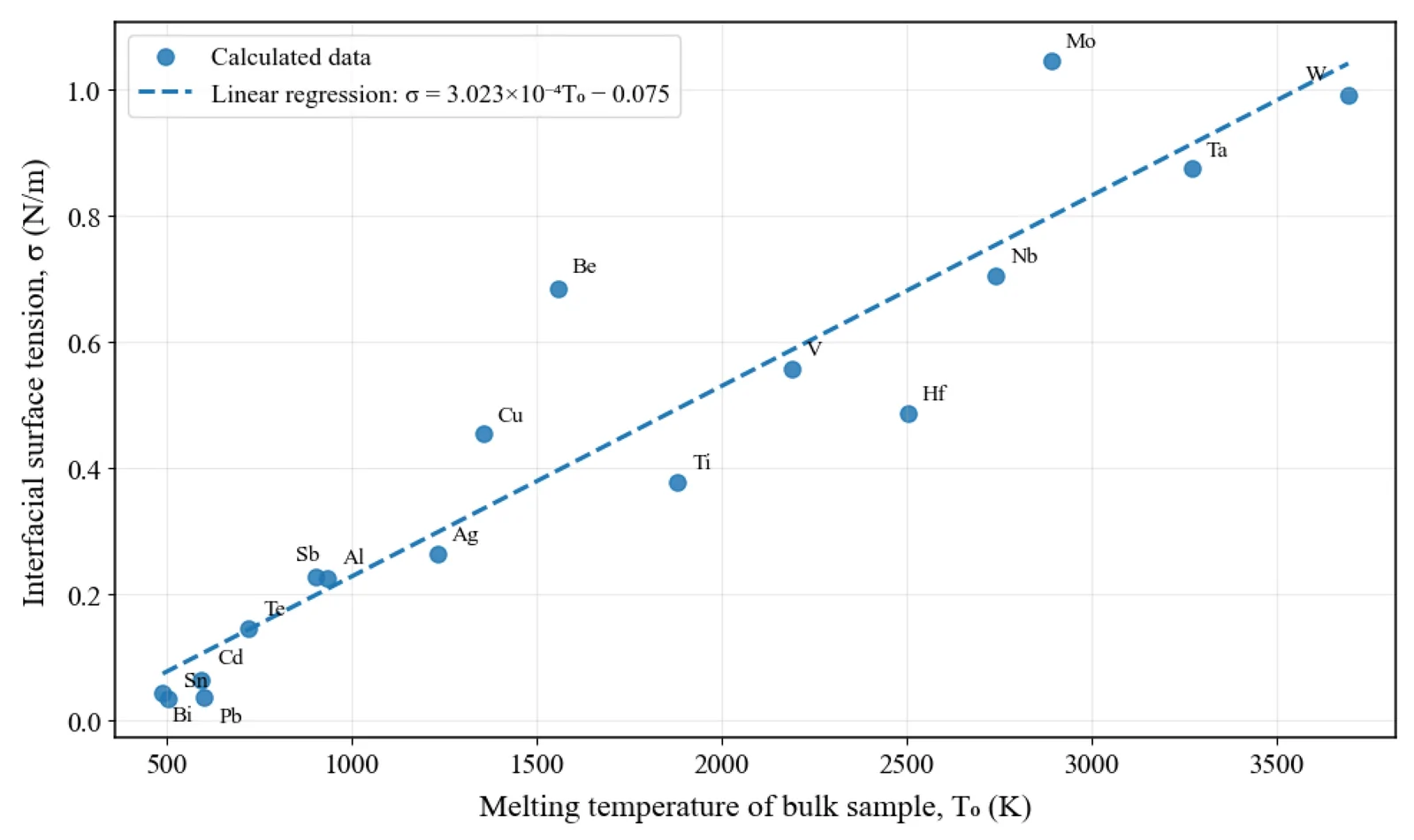
Calculated crystal–melt interfacial tension as a function of the bulk melting temperature.

The calculated interfacial tension generally increases with the bulk melting point and spans nearly two orders of magnitude. It is noteworthy that the value obtained for lead (∼0.035 N/m) is close to the value calculated in Ref. [35] and to the experimental value reported in Ref. [36] (0.04 N/m). As shown in Figure 3, the dependence on bulk melting temperature is approximately linear and is described by $\sigma_{\mathrm{Me}}=3\times 10^{-4}T_{0}-0.075$. The largest deviations from this linear dependence are observed for beryllium, hafnium, and molybdenum and may reflect uncertainties in the heat-of-fusion values.

Using Equation (1), the coefficient *k* was estimated, the melting-point depression was calculated, and interfacial-tension values corresponding to an effective size of 100 nm were determined. The resulting linear approximations of $\sigma_{\mathrm{Me}}$ over the interval from $d_{\mathrm{Me}}$ to 100 nm [30] are given in Table 3. The non-zero values of the coefficient *A* formally indicate a residual size dependence in these linear approximations. Because the experimental data exhibit substantial scatter and the extrapolation is approximate, the calculated interfacial tension is described here as only weakly size-dependent within the accuracy of the present treatment, rather than as strictly size-independent.

## 4. Conclusions

In this study, 34 binary systems were investigated using controlled co-sputtering of dissimilar metals. The experimentally determined parameter $d_{\mathrm{Me}}$ is interpreted as the nominal thickness of a single deposited metal sublayer rather than as a directly measured nanoparticle diameter. The concentration of the second component for each binary system is reported together with the corresponding characteristic sublayer thicknesses.

A formal linear fit between the characteristic sublayer thickness and the bulk melting temperature gives $d_{\mathrm{Me}}=1.46-7.3\;\times 10^{-5}T_{0}$. The low coefficient of determination, $R^{2}=0.007$, shows that the bulk melting temperature alone explains only a small fraction of the observed variation. The characteristic conditions of alloy formation also depend on composition and deposition parameters. Equation (3) is therefore interpreted as an approximate empirical trend rather than as a universal law.

The transition to solid-solution formation was identified experimentally by varying the sublayer thicknesses and monitoring the phase composition by X-ray diffraction. At larger sublayer thicknesses, diffraction patterns characteristic of the individual metals were observed, whereas at the characteristic threshold a solid-solution lattice was detected. These observations are consistent with enhanced atomic mobility and a possible quasi-liquid state, although they do not constitute direct proof of such a state and do not exclude other mixing or diffusion mechanisms.

Using the Thomson equation within an effective-size approximation, the crystal–melt interfacial tension was estimated for the constituent metals. The calculated values are approximately described by $\sigma_{\mathrm{Me}}=3\times 10^{-4}T_{0}-0.075$. The additional extrapolation over the interval from $d_{\mathrm{Me}}$ to 100 nm gives non-zero linear slopes; therefore, the calculated interfacial tension is described as weakly size-dependent within the accuracy of the present approximate treatment rather than as strictly size-independent.

The experimentally determined sublayer-thickness parameters and the corresponding model-based thermodynamic estimates provide a practical basis for selecting deposition conditions in related binary systems. Determination of the crystal–melt interfacial tension for dissimilar-element pairs requires a separate study.

## Figures and Tables

**Table 1:** Nominal metal sublayer thicknesses at which alloys were formed and concentration of the second element (B) in the resulting systems.

No.	Alloy A–B	$d_{A}$, nm	$d_{B}$, nm	$C_{B}$, at.%	No.	Alloy A–B	$d_{A}$, nm	$d_{B}$, nm	$C_{B}$, at.%
1	Ta–Cd	1.7	2.1	61.3	18	Nb–Cd	2.1	1.9	84.2
2	Ta–Cd	1.2	2.6	66.2	19	Nb–Cd	2.2	3.2	83.7
3	Ta–Cd	0.2	4.0	74.4	20	Nb–Cd	0.6	1.3	35.5
4	Ta–Al	0.8	1.1	34.8	21	Nb–Cd	2.1	0.4	13.8
5	Ta–Be	1.1	0.2	26.8	22	Hf–Cd	1.8	0.9	5.8
6	Ta–Be	1.3	0.2	23.8	23	Hf–Cd	0.7	0.9	49.7
7	W–Pb	2.4	1.9	28.6	24	V–Pb	0.2	0.3	24.9
8	W–Nb	0.5	0.9	41.9	25	V–Pb	0.4	0.1	13.2
9	W–Nb	1.3	2.4	61.5	26	V–Pb	0.8	0.1	6.0
10	W–Cd	0.7	1.3	68.6	27	V–Pb	0.4	0.1	13.2
11	Mo–Cd	1.5	2.2	52.4	28	V–Cd	0.6	0.3	26.5
12	Mo–Cd	1.7	0.3	13.1	29	Ti–Al	0.7	0.1	20.0
13	Nb–Sn	1.0	0.4	25.5	30	Cu–Ag	1.0	1.0	100
14	Nb–Be	1.0	0.2	22.8	31	Pb–C	1.2	0.1	93.2
15	Nb–Al	1.0	0.4	28.2	32	Pb–Sb	2.9	1.0	11.9
16	Nb–Cu	1.0	1.5	9.0	33	Pb–Bi	1.9	2.0	23.1
17	Nb–Ti	0.8	0.7	48.5	34	Te–Cd	0.7	0.8	69.5

**Table 2:** Calculated crystal–melt interfacial tension of the constituent metals.

Metal	Melting Point, K	$\Delta H_{\mathrm{mp}}$, kJ/mol	$\Delta H_{\mathrm{mp}}$, kJ/kg	Density, kg/$m^{3}$	σ, N/m
Bi	490	11.00	52.637	9780	0.04
Sn	505	7.20	60.652	5850	0.03
Cd	594	6.20	55.155	8650	0.06
Pb	600	4.77	23.021	11,336	0.03
Te	723	17.50	137.147	6240	0.14
Sb	904	20.10	165.079	6691	0.22
Al	933	10.80	400.297	2689	0.22
Ag	1233	11.30	104.756	10,500	0.26
Cu	1356	13.00	204.563	8960	0.45
Be	1560	13.00	1442.841	1848	0.68
Ti	1881	15.10	315.438	4505	0.37
V	2190	17.60	345.505	5960	0.55
Hf	2503	23.90	133.901	13,310	0.48
Nb	2742	28.00	301.367	8570	0.70
Mo	2893	36.00	375.235	10,220	1.04
Ta	3269	35.00	193.424	16,654	0.87
W	3693	35.10	190.927	19,350	0.99

**Table 3:** Calculated crystal–melt interfacial tension over the nominal sublayer-thickness interval from $d_{\mathrm{Me}}$ to 100 nm.

Metal	*A*, N/(m·nm)	*B*, N/m	Metal	*A*, N/(m·nm)	*B*, N/m
Bi	$-7.4\times 10^{-4}$	0.0746	Be	$-4.0\times 10^{-3}$	0.4025
Sn	$-7.9\times 10^{-4}$	0.0792	Ti	$-5.0\times 10^{-3}$	0.5007
Cd	$-1.0\times 10^{-3}$	0.1065	V	$-5.9\times 10^{-3}$	0.5953
Pb	$-1.0\times 10^{-3}$	0.1083	Hf	$-6.9\times 10^{-3}$	0.6910
Te	$-1.4\times 10^{-3}$	0.1460	Nb	$-7.6\times 10^{-3}$	0.7640
Sb	$-2.0\times 10^{-3}$	0.2015	Mo	$-8.1\times 10^{-3}$	0.8102
Al	$-2.1\times 10^{-3}$	0.2104	Ta	$-9.2\times 10^{-3}$	0.9251
Ag	$-3.0\times 10^{-3}$	0.3023	W	$-1.0\times 10^{-2}$	1.0545
Cu	$-3.4\times 10^{-3}$	0.3400	–	–	–

## Data Availability

The data that support the findings of this study are available from the corresponding author upon reasonable request.
